# Experimental Investigation of NaOH and KOH Mixture in SCBA-Based Geopolymer Cement Composite

**DOI:** 10.3390/ma13153437

**Published:** 2020-08-04

**Authors:** Sardar Kashif Ur Rehman, Lahiba Imtiaz, Fahid Aslam, Muhammad Khizar Khan, Muhammad Haseeb, Muhammad Faisal Javed, Rayed Alyousef, Hisham Alabduljabbar

**Affiliations:** 1 Department of Civil Engineering, COMSATS University Islamabad, Abbottabad Campus, Abbottabad 22060, Pakistan; muhammadkhizar.khan@ugent.be (M.K.K.); muhammadhaseebd044@gmail.com (M.H.); arbabf1@gmail.com (M.F.J.); 2 Department of Civil Engineering, College of Engineering in Al-Kharj, Prince Sattam bin Abdulaziz University, Al-Kharj 11942, Saudi Arabia; f.aslam@psau.edu.sa (F.A.); h.alabduljabbar@psau.edu.sa (H.A.); 3 Department of Civil Engineering, Ghent University, B-9052 Gent, Belgium

**Keywords:** activators, durability, geopolymer cement, molarity, sugar-based bagasse ash (SCBA)

## Abstract

This research aimed at exploring the effects of a mixture of sodium hydroxide (NaOH) and potassium hydroxide (KOH) activators in a sugar cane bagasse ash (SCBA)-based geopolymer cement paste. Bagasse ash replacement was 20% of cement by weight. The mixture of NaOH and KOH comprised 4, 8, and 12 M solutions with mixing percentages of 0%, 20%, 40%, 60%, 80%, and 100% for all possible combinations. A pH test was performed on each possible combination of solutions. A Chapelle’s test, XRD, X-ray fluorescence (XRF), and SEM analysis were used to check whether the SCBA exhibited pozzolanic reactivity. Subsequently, the SCBA geopolymer cement paste was tested for compressive strength, water absorption, permeable porosity, and sorptivity. It was estimated that the geopolymer cement paste exhibited higher absorption and sorptivity values than control mixtures when molarity increased. However, the samples prepared with combinations of the 8 M activator solution exhibited consistent absorption, sorptivity, and compressive strength values when compared to the control and other geopolymer mixtures with 4 and 12 M activator solutions. Thus, the two activator solutions G8N_40_8K_60_ and G8N_20_8K_80_—where GxN_a_yK_b_ represents the geopolymer concrete sample prepared by adding solutions of two bases, i.e., ‘xN_a_yK_b_’ showing an ‘a’ percentage of ‘x’ molar NaOH and a ‘b’ percentage of ‘y’ molar KOH—were obtained as the optimum molar ratio of the activator in geopolymer concrete. The geopolymer cement pastes, along with the optimum and control samples, were further tested for concrete durability, SEM, and TGA tests. The G8N_20_8K_80_ sample exhibited a better mechanical and durability performance than the G8N_40_8K_60_ sample. The durability performance of the geopolymer concrete was also superior to ordinary concrete. Moreover, the geopolymer concrete achieved a 21% reduction in global warming potential compared to the control mixture. Thus, it can be concluded that the use of SCBA in geopolymer concrete can address the ash disposal and CO_2_ emission problems with enhanced durability.

## 1. Introduction

A constant increase in the demand of infrastructure is leading to the rapid economic growth of any country. Concrete is the most widely used construction material in the construction industry due to its variable substantial properties. However, its conventional production makes use of ordinary Portland cement (OPC), which emits CO_2_, nitrogen oxides, and sulphur oxides, and it exhausts natural resources. The emission of CO_2_ and the requirement of energy in the production of OPC hampers sustainability. Previous research has claimed that cement manufacturing emits an almost equal quantity of CO_2_ into the atmosphere [1]. About 7% of the total CO_2_ emissions in the atmosphere are due to cement production, thus causing environmental pollution [2]. On the other hand, the removal of industrial waste and agricultural resources such as fly ash (FA), slag (SG), red mud mine tailing, rice husk ash (RHA), silica fume, and palm oil is a major ecological and environmental issue. The uncontrolled burning of these ashes for energy production has also led to a disposal problem. Thus, the management and disposal of these wastes are becoming big challenges. Sugar cane bagasse ash (SCBA) is the combustion byproduct of sugar boilers and alcohol factories. Pakistan is ranked fifth in the production of sugar cane worldwide, with an approximate yearly yield of 79.9 million tons [3]. Bagasse production is approximately 1.46 million tons every year in Pakistan. Each ton of sugar cane produces about 26% of its weight in bagasse, which is used as a source of energy that subsequently leads to 0.62% of its weight in residual ash, thus inducing a large amount of disposed ash. The introduction of geopolymer concrete technology has led to sustainable development. As per [4], the production of geopolymer concrete with landfilled waste has a lower impact on global warming than standard OPC concrete. Therefore, this research work accomplished multiple objectives: reducing ash disposal problem and CO_2_ emissions and helping in the sustainable production of cement composites.

SCBA is used as an alternate supplementary cementitious material and as a mineral admixture in concrete and mortar due to the presence of a large amount of amorphous silica. It has been used to improve both the durability and strength properties of concrete when partially replaced with cement [5]. Shukla et al. [6] studied the effect of bagasse ash as a partial replacement of cement, and they concluded that bagasse ash provides its maximum compressive strength at the partial replacement of 10% of cement due to its pozzolanic properties. Moreover, Ganesan et al. [7] examined the physical and mechanical properties of hardened concrete partially replaced with SCBA. They found that SCBA is an effective and optimal mineral admixture, with 20% as the most favorable cement replacement ratio. Geopolymer concrete comprises the class of reactive aluminosilicates such as metakaolin, SG, RHA, FA, and high calcium wood ash activated with silicate solution or an alkali metal hydroxide solution by the polymerization process. SCBA is an eco-friendly alternative binder to OPC that significantly reduces CO_2_ emissions and provides higher resistance in aggressive environments [8,9]. Vignesh et al. [10] investigated the strength parameters of FA-based geopolymer concrete with ground granulated blast furnace slag (GGBS). They found that geopolymer concrete is a modern industrialized material and a real alternative to OPC that attains its compressive strength via the polycondensation of silica, alumina, and its high alkali content [11]. However, when combined with OPC, it increases its compressive strength due to the presence of calcium silicate hydrate (CSH) and the polycondensation of silica and alumina [12].

Mostly, earlier studies were conducted on FA-based geopolymer concrete. Later, SCBA blended with RHA and with other aluminosilicates were used to produce geopolymer concrete. Many recent studies have also been conducted on binary mixtures of FA–SCBA and blast furnace slag (BFS) mixed with SCBA by using alkali hydroxides and silicates mixtures, mainly (Na or K) alkaline activators [13]. However, per the author’s knowledge, no systematic study to describe the properties of a geopolymer concrete consisting of SCBA only activated with the combination of two activators, such as NaOH and KOH, has yet been conducted. Furthermore, what concentration of the blend of NaOH and KOH would effectively activate an SCBA-based geopolymer concrete? What would be the durability performance of an optimum SCBA-based geopolymer concrete? These questions are still to be answered. Therefore, an SCBA-based geopolymer concrete activated with a blend of NaOH and KOH was investigated to achieve the optimum percentage of strength and other desired properties.

## 2. Experimental Investigation

### 2.1. Materials

#### 2.1.1. Cement

OPC from the Bestway cement factory, per American Society for Testing and Materials (ASTM) international standards, ASTM C 150 Type I, was used throughout the study. The cement’s fineness was found to be 2670 cm^2^/gm by the Blaine air permeability apparatus (ELE International, Bedfordshire, UK). Chemical composition of cement is given in Table 1.

#### 2.1.2. SCBA

SCBA was acquired by open burning. It was sieved through a #50 sieve to remove both coarse and fine fibrous carbon particles [14]. SCBA was then subjected to grinding for 120 min to achieve its maximum pozzolanic activity [15]. Its fineness was found to be 2863 cm^2^/gm after grinding for 120 min.

#### 2.1.3. Mixing Water

Distilled water obtained from the environmental lab was used throughout the experimental work.

#### 2.1.4. Coarse and Fine Aggregate

Coarse and fine aggregates were brought from Abbottabad. The sieve analysis was conducted as per ASTM C136–04 [16]. The physical properties of the aggregate are given in Table 2.

#### 2.1.5. NaOH and KOH Activator

KOH and NaOH were obtained from the Sigma-Aldrich (Karachi, Pakistan). Activator solutions of 4, 8, 12, and 16 M NaOH and KOH were prepared by dissolving the specified quantity in distilled water. For instance, the ‘X’ molar NaOH solution was prepared by dissolving (40*X) grams in one liter of distilled water, and the ‘X’ molar KOH solution was prepared by dissolving (56*X) grams in one liter of distilled water. The different molar solutions were subjected to pH tests.

### 2.2. Testing Program

Testing program consists of following three phases.

Phase 1 (characterization of Bagasse ash and different molar solutions of NaOH and KOH activators): For SCBA characterization; XRD (model Ringaku Mini Flex, 600, Tokyo, Japan), SEM (Hitachi TM 3030 tabletop microscope, Japan), X-ray fluorescence (XRF) (JSX-1000S XRF, Japan), and Chapelle and Blaine air permeability tests were carried out after sieving and then grinding, while pH tests for the blend of different molar solutions of NaOH and KOH were also conducted.

Phase 2 (testing and analysis of the SCBA-based geopolymer cement paste): The SCBA-based geopolymer cement paste was prepared by mixing different combinations of molar solutions of NaOH and KOH in the mixtures containing cement with 20% replaced bagasse ash and a water to binder ratio of 0.5. The detailed mix design is presented in Table 3 The prepared mixtures were cast in 5 × 5 × 5 cm mortar cubes’ molds, and after 24 h, the samples were demolded and immersed in curing tank for 7 and 28 days. The testing involved a compression test, a water absorption test, a sorptivity test, and a permeable-porosity test at the 7 and 28 day curing stages.

Phase 3 (testing and analysis of the SCBA-based geopolymer concrete sample): The geopolymer concrete sample was cast in 150 × 150 × 150 mm concrete cubes and cured for 7 and 28 days in a curing tank. The mix design for the concrete samples is tabulated in Table 4. The optimum chosen samples from phase 2 were further tested for a slump test, a concrete durability test, and a TGA test along with the tests involved in phase 2. Furthermore, the temperature behavior of molar solutions was analyzed by casting the same geopolymer cement paste cubes at different conditions.

#### 2.2.1. Specimen Designation

In this experimental investigation, the various mixtures were abbreviated in different forms, namely CM_,_ BM, GxNyK, and GxN_a_yK_b_. The specimen cast without the addition of bagasse ash was termed CM (control mixture of cement), the specimen cast with 20% bagasse ash replacement was designated BM (control bagasse ash mixture), and GxN_a_yK_b_ represents the geopolymer concrete sample prepared by adding solutions of two bases, i.e., ‘xN_a_yK_b_’ showing an ‘a’ percentage of ‘x’ molar NaOH and a ‘b’ percentage of ‘y’ molar KOH. For instance, the designation G4N_20_8K_80_ indicates the geopolymer concrete sample prepared from the activator solution of a 20% 4 M NaOH and 80% 8 M KOH mixture.

#### 2.2.2. Testing of SCBA (Phase 1)

A Chapelle’s test was conducted in accordance with French Norm NF P18-513 [17] to assess the existence of pozzolanic activity of ground SCBA, which was mainly determined by the consumption of Ca(OH)_2_. It has been observed that the amount of silica present in ash plays a crucial role in pozzolanic activity [18]. Therefore, XRF was performed to check the chemical composition of the SCBA produced after grinding. However, the mineralogical characteristics were determined with the aid of XRD. The chemical phases from the diffraction pattern of SCBA were identified using the “MATCH Phase Identification v3.1” software. Additionally, SEM analysis was conducted on ground SCBA at different magnifications ranging from ×500 to ×3500 to check its morphology.

#### 2.2.3. Testing on Geopolymer Cement Paste (Phase 2)

The compressive strength test was performed as per ASTM C39/C39M–18 [19]. The water absorption test was performed as per ASTM C642-97 [20] to experimentally determine the water absorption capacity for different samples of the geopolymer cement paste. A sorptivity test was used to measure capillary water absorption of the SCBA-based geopolymer paste as per ASTM C1585-13 [21], while a permeable porosity test was conducted according to ASTM C642-06 [22]. The permeable porosity test was based on the apparent volume of permeable voids (AVPV). Water absorption was measured by manipulating the differences in specimen weight under fully saturated and oven-dried conditions, as follows:

(1) Water absorption, % =(Ws−Wi)/Wi×100

where W_s_ is the weight of the sample in the saturated state and W_i_ is the initial weight of the sample after oven drying.

The volume of permeable voids was calculated as:

(2) $$\mathrm{Volume}\;\mathrm{of}\;\mathrm{permeable}\;\mathrm{voids},\;\%\;=\left(g_{2}-g_{1}\right)/g_{2}\times 100$$

where

(3) $$\mathrm{Apparent}\;\mathrm{density}=\;g_{2}\;=\left(\frac{Wi}{Wi-Ww}\right).\;\rho$$

(4) $$\mathrm{Bulk}\;\mathrm{density},\;\mathrm{dry}=\;g_{1}\;=\left(\frac{Wi}{Wb-Ww}\right).\rho$$

where *W_b_* is the weight of the boiled sample after leaving for 5 h in boiling water and *W_w_* is the apparent weight of the sample after leaving for 5 h in boiling water

#### 2.2.4. Testing on Geopolymer Concrete (Phase 3)

In phase 3, the tests conducted in phase 2 were followed with the same standards on the chosen optimum geopolymer concrete specimens. However, further tests like the slump test, the acid attack test, and TGA were conducted at 7 and 28 days of curing. In the slump test, the standard slump cone apparatus (National Scientific Corporation, Lahore, Pakistan) was used for each control and geopolymer concrete sample; ASTM C143 was used to determine slump values. A digital Vernier caliper (National Scientific Corporation, Lahore, Pakistan) was employed to record the slump values. TGA was performed according to ASTM E1131–03 [23] to determine the change in weight of the cement paste containing SCBA as the function of temperature or time under a controlled environment. The cement paste was soaked in acetone to stop the hydration reaction, dried, and then ground. TGA was conducted in a nitrogen atmosphere up to 800 °C at a uniform heating rate of 10 °C/min. Moreover, an acid attack test was performed according to ASTM C267 [24], as this is the standard test method to find the chemical resistance of mortars, grouts, and polymer concretes [25]. It is noteworthy to mention that three samples of each mixture were prepared and tested under the same experimental conditions, and the average value is reported. The variation of the individual result was kept at less than 5% of the average value; otherwise, the mixtures were cast again.

## 3. Test Results and Discussion

### 3.1. Phase 1 Test Results

#### 3.1.1. Chapelle’s Test

The minimum requirement for the pozzolanic activity is 330 mg of CaO/g of pozzolan due to the presence of amorphous silica active in nature [26]. The test results of the Chapelle’s test of SCBA are represented in Table 5, showing a 310.7% increase of reactivity over the standard. Hence, it can be concluded that SCBA is highly reactive.

#### 3.1.2. XRD and XRF Analysis

**Figure 1:**
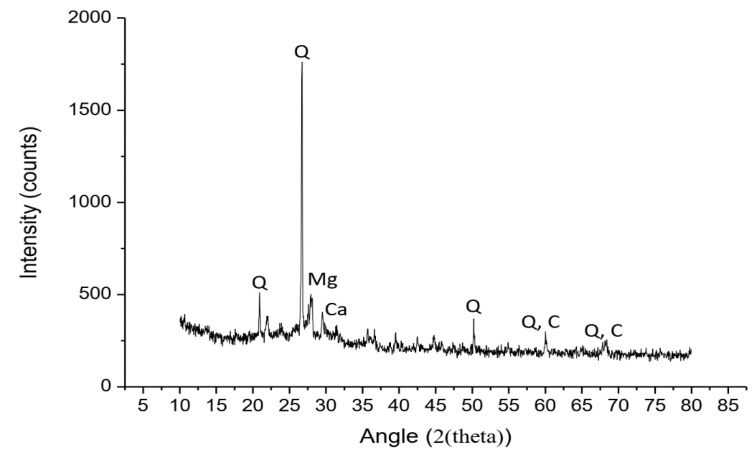
XRD analysis of SCBA.

An XRD analysis of SCBA was conducted using the MATCH Identification software, and the results are presented in Figure 1. The MATCH Identification software analyzed the powder diffraction data and compared them with reference patterns for phase identification. The XRD pattern showed that there was only one large peak of quartz, along with small peaks of cristobalite and calcite (Ca). The quartz (Q) had a hexagonal axes crystal system with maximum peak of 26.2° at 2θ, whereas the Ca and cristobalite (C) exhibited a tetragonal crystal system with quantities of 7.9% and 5.9%, respectively. The calcite showed its small peak at 2θ of 28°. The cristobalite belongs to a group with a different crystal structure than quartz, thus resulting in its small peaks. It is known that the presence of a higher number of larger peaks represents the crystal nature of ash. However, the reactive properties in the ash sample were only due to the presence of amorphous silica because it is more reactive and desirable for greater pozzolanic activity than crystalline silica [27]. However, the XRD pattern contained a higher number of small peaks, except for one larger peak of quartz. Thus, it can be concluded that SCBA mostly consisted of amorphous silica after being sieved from #50 sieve and being ground for 120 min.

According to ASTM C618, a material is classified as the pozzolan if the percentage of silica SiO_2_, alumina Al_2_O_3_, and iron oxide Fe_2_O_3_ is greater than 70%. The XRF analysis of SCBA is presented in Table 6. The test results indicated that SCBA is a reactive pozzolan with a percentage of silica, alumina, and iron oxide of 85.87%, thus meeting the minimum requirement of ASTM C618.

#### 3.1.3. SEM Analysis

**Figure 2:**
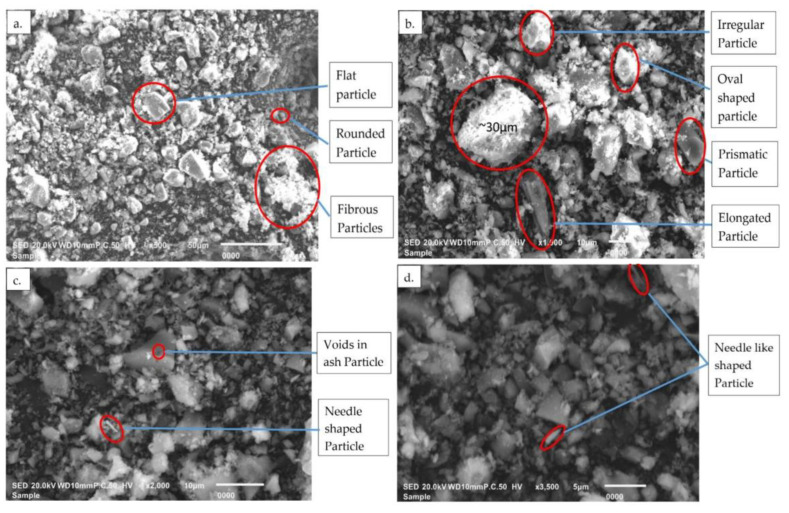
SEM image of ground SCBA (**a**) at 500 magnification, (**b**) at 1000 magnification, (**c**) at 2000 magnification, and (**d**) at 3500 magnification.

SEM images of the SCBA are shown in Figure 2. The images show a number of shapes like elongated, rounded, prismatic, needle-like, and irregular. The flat, rounded, and fibrous particles are displayed in Figure 2a, while the elongated, prismatic, oval-shaped, and irregular particles are shown in Figure 2b. It is known that the presence of irregular particles is representative of the silica-rich nature of SCBA [14]. Conversely, the presence of rounded and spherical particles in SCBA is due to melting at high temperatures, while the presence of fibrous particles represents the carbon content in ash [14]. It was further observed that the ash exhibited large particles of approximately 30 µm. However, most particles were smaller than 5 µm due to the high surface area of ground ash. The needle-shaped particles and the minute number of voids in the ash particles are represented in Figure 2c,d at the higher magnifications of ×2000 and ×3500. The small number of voids in SCBA may have been due to the uncontrolled burning and grinding of the sieved ash. However, the presence of a large number of voids indicated the greater water absorption of the mixture containing SCBA [28].

#### 3.1.4. pH Test Results

**Figure 3:**
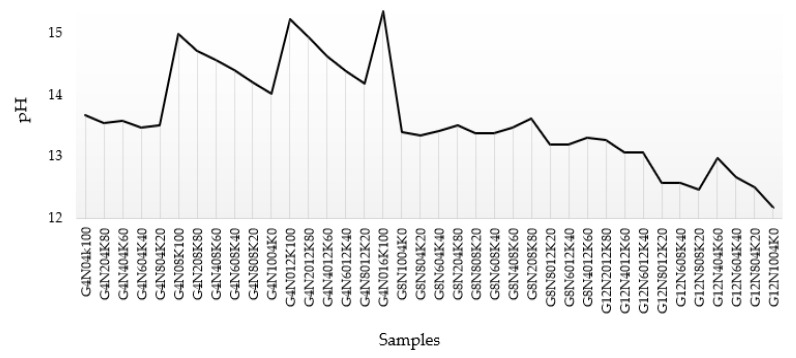
pH values of geopolymer samples.

The pH test was performed with a pH meter to check the hydrogen ion activity of the blend of NaOH and KOH solutions. A total of 38 solution samples of specified molarity were prepared by mixing pellets in distilled water with a magnetic stirrer. Figure 3 represents the chart that shows the pH ranging from 12.17 to 15.35. The sample G4N_01_6K_100_ exhibited the maximum value of pH due to the greater molarity of the strong base KOH.

### 3.2. Phase 2 Test Results

#### 3.2.1. Water Absorption Test

**Figure 4:**
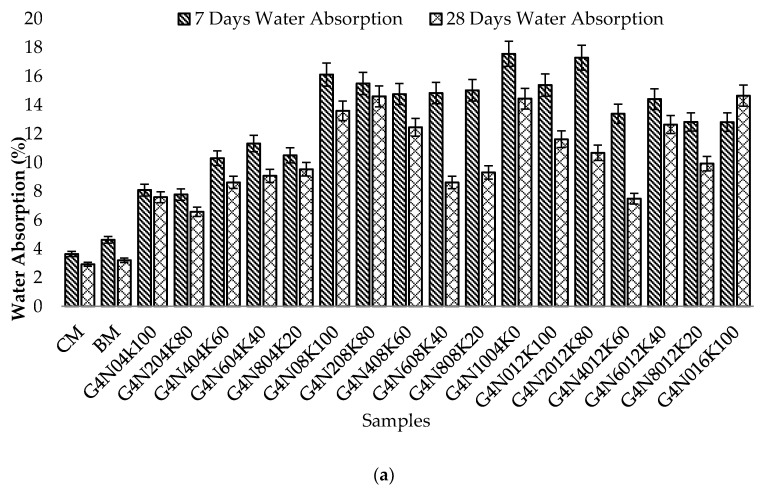

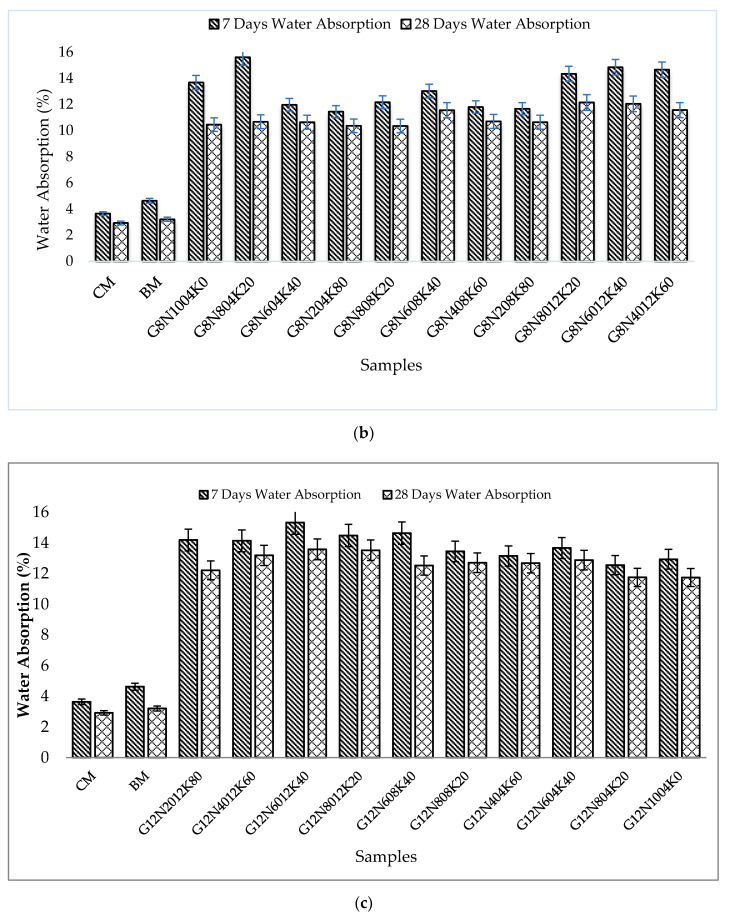
Water absorption of geopolymer cement paste at 7 and 28 days: (**a**) 4 M samples, (**b**) 8 M samples, and (**c**) 12 M samples.

The water absorption test results of all geopolymer paste samples are given in Figure 4a–c with varying percentages of 4, 8, and 12 M NaOH and KOH, respectively. Contrary to expectations, a comparison of all geopolymer samples revealed that there was a general rise in water absorption with increasing molarity. The behavior was due to higher temperature release with the molarity increase, which resulted in small cracks, thus causing more absorption [29]. However, these cracks could be reduced by using admixtures. Additionally, it was found that alkali enrichment caused pores in the cementitious system that further resulted in shrinkage cracks. These cracks and pores caused an increase in the absorption of geopolymer samples [30]. The increasing trend rose significantly in the 4 and 12 M blend solution samples. However, in the case of 8 M solution samples, the rising trend was not very significant, representing the blend with consistent water absorption values. This behavior could be attributed to consistent permeable air voids in the 8 M samples. It was also observed that the samples cast with higher molarity of KOH had greater absorption values due to such values’ dependence on cat-ion type and pH values [31,32,33]. Furthermore, the seven day water absorption values of the geopolymer paste samples were more than those of the 28 day values. This was due to the incomplete polymerization process at the earlier age.

#### 3.2.2. Sorptivity Test

**Figure 5:**
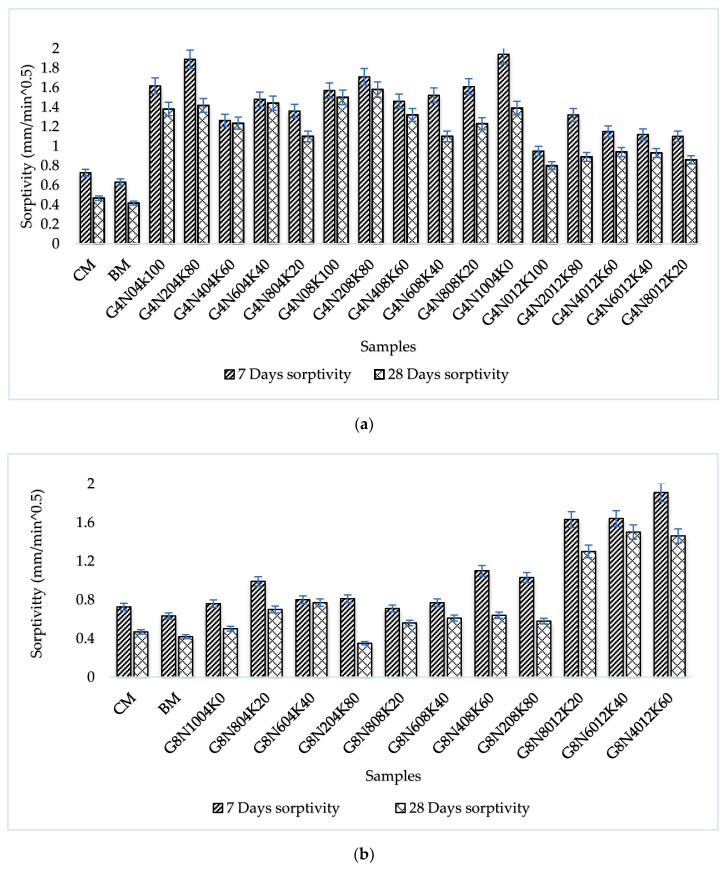

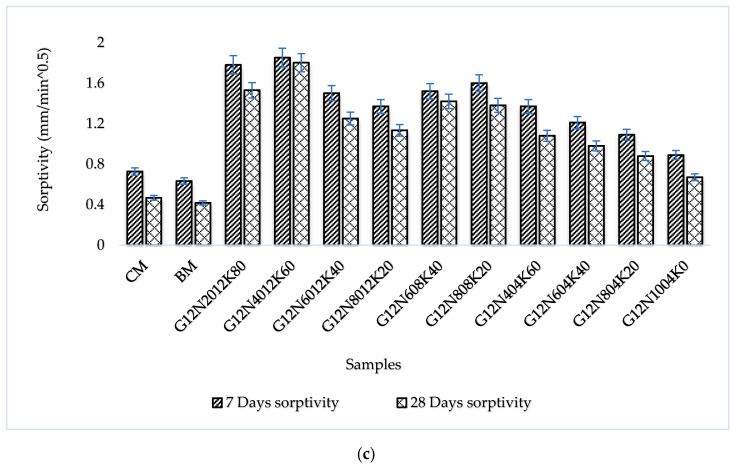
Sorptivity values of geopolymer cement pastes at 7 and 28 days: (**a**) for 4 M samples, (**b**) for 8 M samples, and (**c**) for 12 M samples.

**Figure 6:**
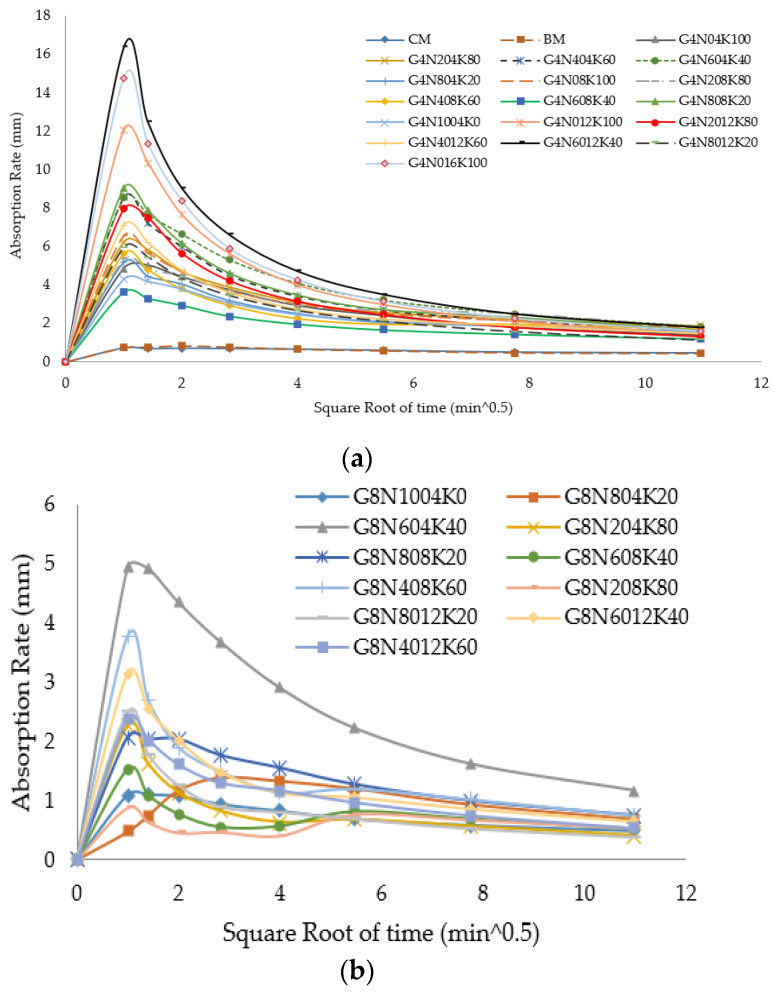

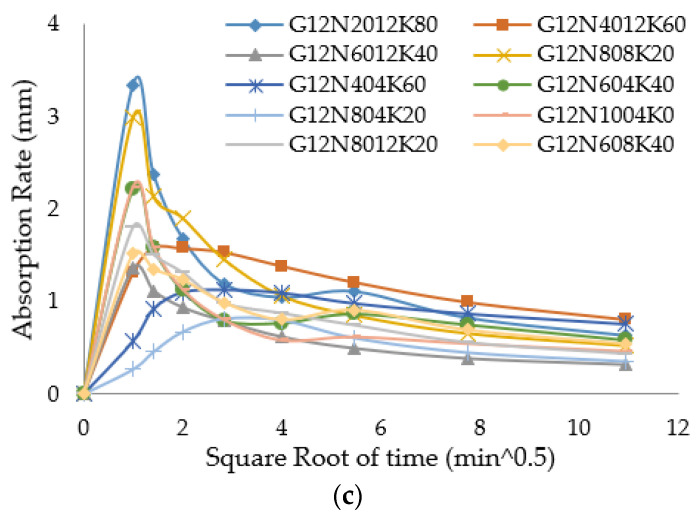
Absorption rate of geopolymer cement paste at 28 days: (**a**) for 4 M samples, (**b**) for 8 M samples, and (**c**) for 12 M samples.

Sorptivity represented the capillary rise and moisture transport in the unsaturated part of the concrete sample, and it is recognized as a significant factor for the durability of concrete [34]. The connectivity and permeability of the pore system and its pore size determines the sorptivity and concrete performance in aggressive environments [35]. The sorptivity values for the 4, 8, and 12 M geopolymer cement paste samples at seven and 28 days are given in Figure 5a–c, respectively. The cumulative water absorption versus square root of time values for the 4, 8, and 12 M geopolymer cement paste samples at 28 days are shown in Figure 6a–c. Following to ASTM C-1585, the sorptivity values of each geopolymer sample were calculated as the slope of the sorptivity curve. The sorptivity results showed that initially, the capillary rise was higher due to the capillary force that controlled initial absorption. Additionally, CM and BM had lesser absorption rates than the geopolymer cement pastes [8]. Sorptivity values were higher in the high molar ratio samples of each blend, showing that the increase in the capillary rise of the geopolymer cement paste samples was due to the presence of microcracks caused by the vigorous exothermic reaction in alkali-enriched environment [36]. Conversely, the micro-cracks may have been due to the presence of shrinkage-induced stresses. However, the effect of the shrinkage on the SCBA-based geopolymer samples was not studied in this research work. Additionally, the results showed the higher sorptivity values of the seven day geopolymer samples than the 28 day samples due to the incompletion of both the CSH phase and the geopolymeric reaction.

#### 3.2.3. Permeable Porosity Test

**Figure 7:**
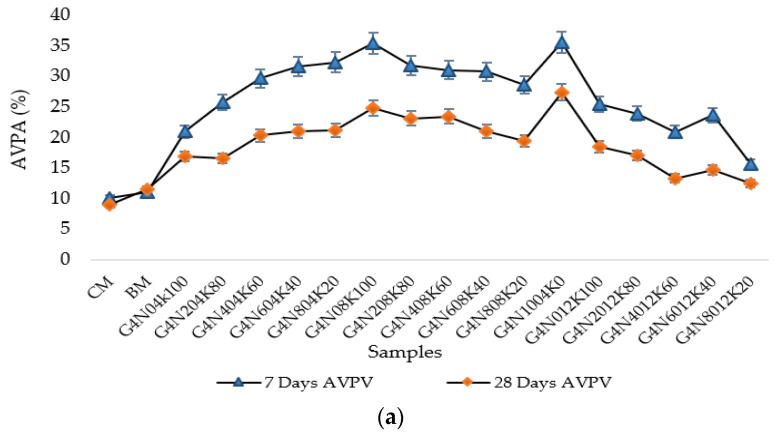

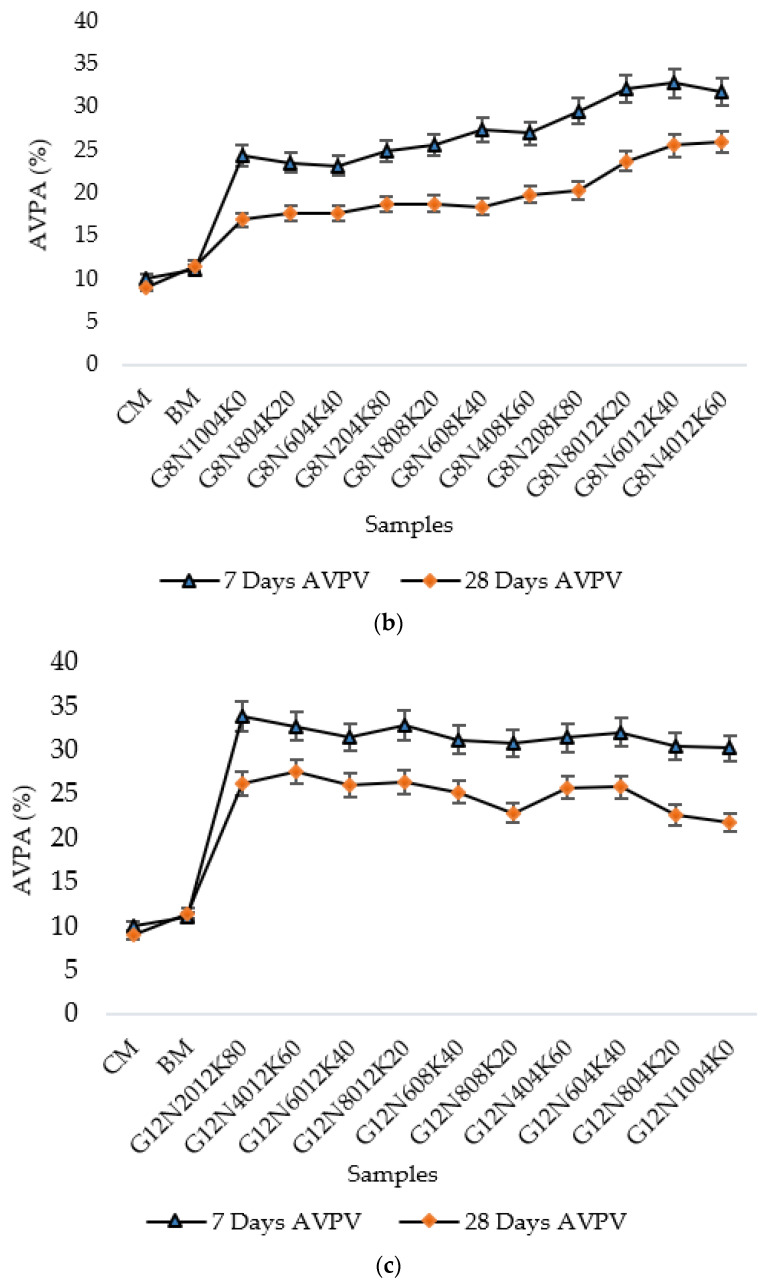
Apparent volume of permeable voids (AVPV) of geopolymer cement pastes: (**a**) for 4 M samples, (**b**) for 8 M samples, and (**c**) for 12 M samples.

**Figure 8:**
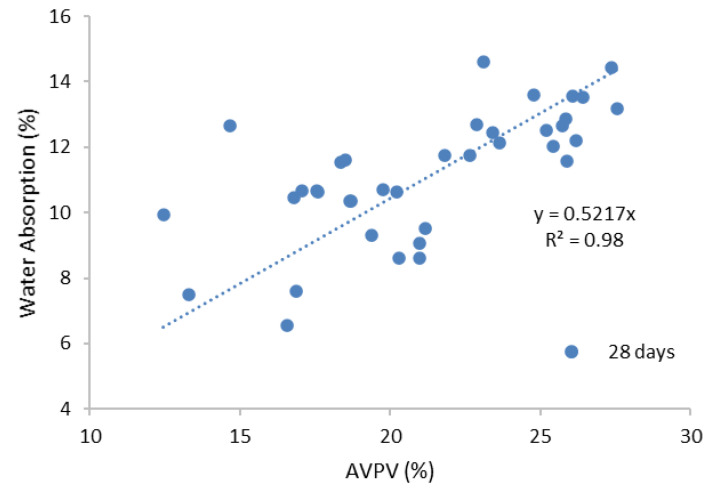
AVPV and water absorption relationship.

The effective permeable porosity of the geopolymer samples depended on water absorption and the AVPV [8]. The AVPV values of the geopolymer samples of 4, 8, and 12 M blends are given in Figure 7a–c. Generally, it can be seen that the AVPV values of the higher molar samples were greater than those of the lower molar samples. This was the same trend as sorptivity and absorption values. It was known that alkali enrichment would cause the early and quick hydration of the system, resulting in a large number of cracks [30]. It was observed that the samples containing 100% NaOH or KOH exhibited higher AVPV values due to the presence of greater voids in the samples. This revealed that the percentages of different molar ratios in the solution affected the mechanical and durability properties of the geopolymer cement pastes. Furthermore, the control samples had lower voids ratios than the geopolymer samples. The difference between seven and 28 day AVPV of the control samples was less than the percentage decrease of the AVPV of the geopolymer samples. This may have been due to the effect of the curing temperature, causing the presence of several voids with enhanced capillary mechanisms [37]. Additionally, the 8 M samples showed an increasing trend with the increase of molarity, while the 12 M samples showed a somehow constant change, suggesting that almost all 12 M samples exhibited higher voids and pores. Lastly, the relationship between AVPV and water absorption is shown in Figure 8, which shows an appreciable coefficient of determination, i.e., 0.98, so the water absorption rose with the corresponding increase in voids.

#### 3.2.4. Compression Test

**Figure 9:**
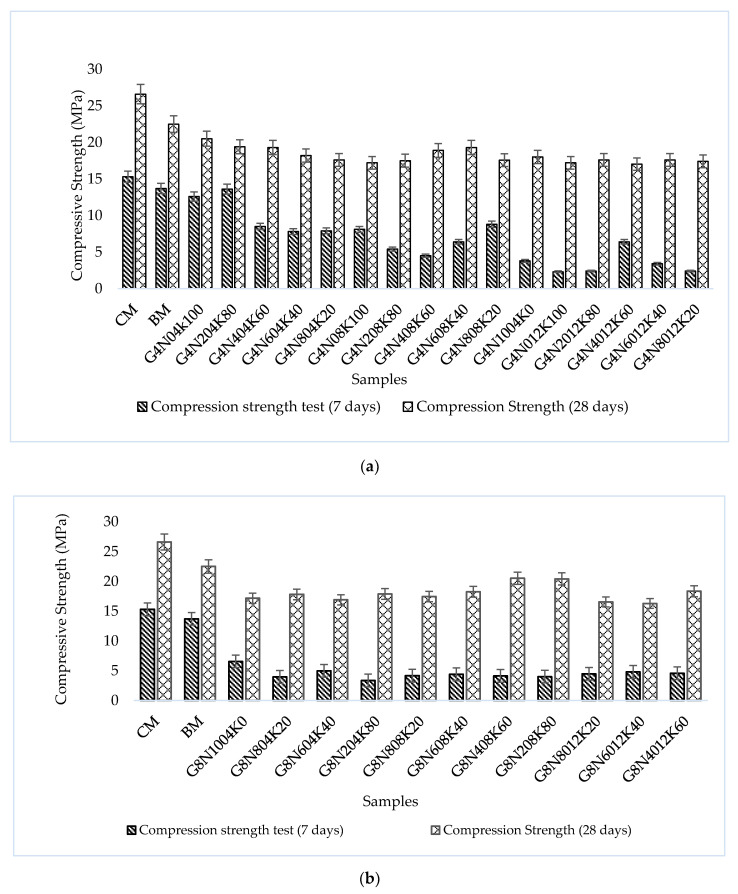

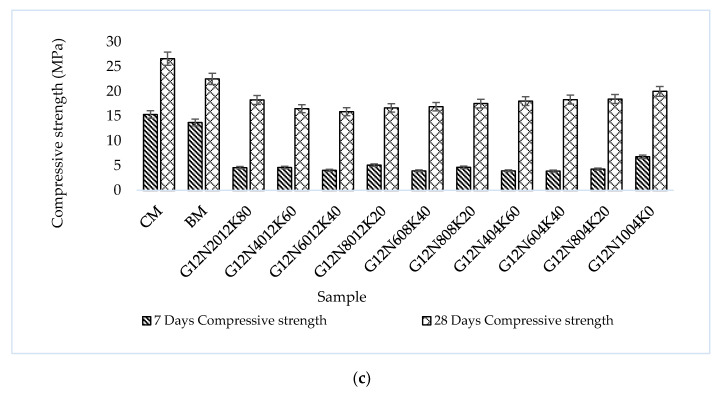
Compressive strength at 7 and 28 days: (**a**) for 4 M samples, (**b**) for 8 M samples, and (**c**) for 12 M samples.

The compressive strength test results of control mixtures and geopolymer samples at seven and 28 days are given in Figure 9a–c. The results showed that the early age strength of the geopolymer samples was less than that of the later age samples because the compressive strength of the geopolymer samples decreased when cured at ambient temperature [38]. Additionally, the polymerization reaction and CSH gel formation at an early age were incomplete, causing a reduction in strength. In practical applications, low strength at early ages can be overcome by the use of a small quantity of additives, i.e., accelerators in GPC to accelerate the early-age reaction. Moreover, curing at elevated temperatures is also beneficial to overcome this problem. Additionally, it was observed that the compression strengths of the 4 and 8 M samples were relatively higher than that of the 12 M sample because of the formation of shrinkage cracks in higher molarity [30]. The sample cast with a 16 molar solution of G4N_0_16K_100_ showed a negligible compressive strength due to high molarity, temperature effects, and shrinkage cracks. It was found that the measurement of strength and shrinkage both depended on the type of Na^+^ and K^+^ ion present in the activator [33].

**Figure 10:**
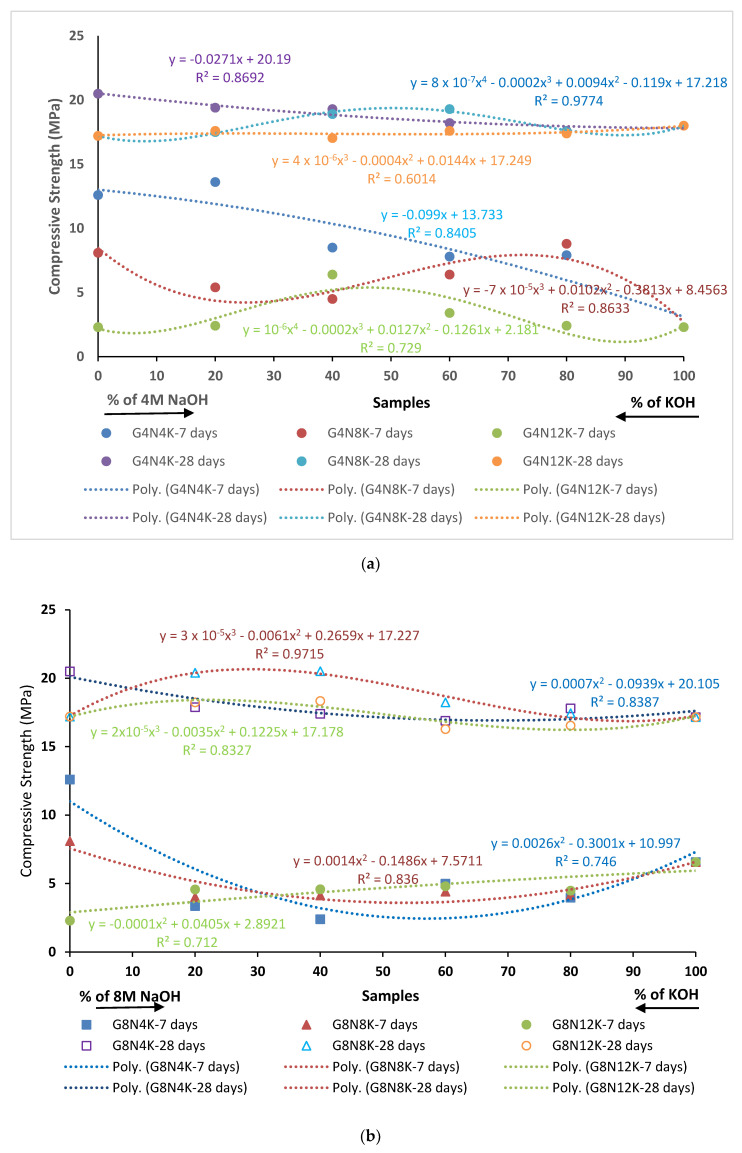

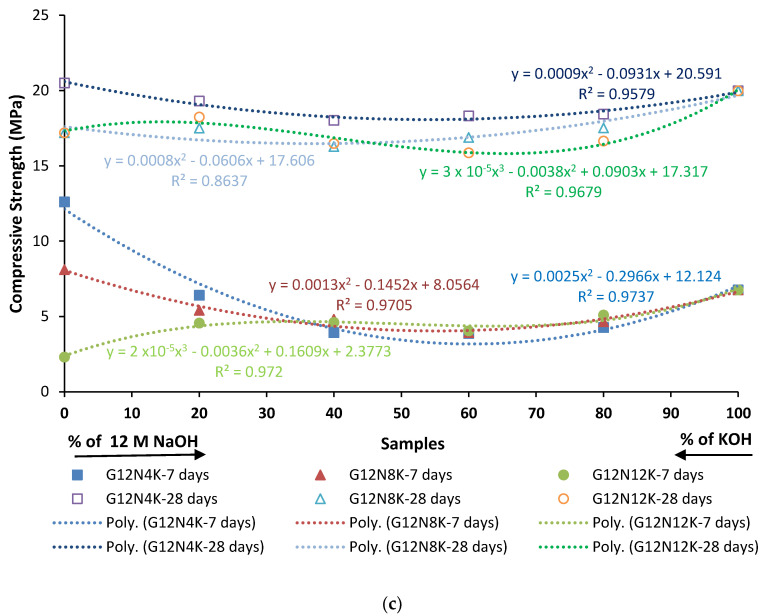
Compressive strength at different percentages of activator solution with respect to activator percentage: (**a**) for 4 M samples, (**b**) for 8 M samples, and (**c**) for 12 M samples.

The changing trend in compression strength concerning the percentage of activators was similar at seven days for samples of all molarities (Figure 10). For instance, the compressive strength increased with a rise in the percentage of 4 M KOH and decreased with a higher concentration of 12 M KOH for all the samples, whereas raising the 8 M KOH percentage showed no change. At 28 days, the rising trend of compression strength could be seen with the incline in the percentage of KOH, except for the 12 M solution, but the rise was not notable when compared to seven day strength. This meant that there was a significant difference in the seven and 28 day strength values, along with the high molarity, in the samples. The high difference in strength could be explained by the considerable additional increase in the strength of the polymeric composites with time, as well as the pozzolanic action of aluminosilicate [39,40].

Though the mixtures showed low strength, they could be used for non-structural applications like in floors, paving blocks, different building layers, ditches, sidewalks, boat ramps, light pavements, and concrete repairs—especially at those places where high mechanical strength is not required [41]. Moreover, use of activators, curing at elevated temperatures, and a strength-based mix design method can significantly enhance the compressive strength of GPC. According to research conducted by Aldred and Day [41], geopolymer specimens exposed to fire fulfill the requirement of the Australian Standard (AS 1530) [42] and can be successfully used in fire-resistant applications, e.g., chimneys and furnaces, in Australia. In addition, they found that geopolymer concrete samples have a very low penetrability of chloride ion and meet = ASTM standards (ASTM C1202) [43], which opens their application in marine exposure sites and coastal protection armors. Furthermore, it was also observed that the durability of GPC was higher than a control mixture [44] and fulfilled the AS 3600 [45] requirements, so this GPC could be used in acid-resistant sewer pipes, water-based non-structural concrete, and sewerage treatment/handling. It is important to mention here that a geopolymer cannot be considered a substitute for cement. Rather, its applications are mainly in those areas where the utilization of cement has certain constraints.

Generally, a non-linear relationship between percentage and compressive strength was found at lower molarities; for example, in the case of G4N_4_K, the coefficients of determination were 0.97 and 0.86 at seven and 28 days, respectively. However, with the increase in molarity, this relationship vanishes, thus depicting the unexpected behavior at higher molarities due to shrinkage cracks and incomplete polymerization.

### 3.3. Phase 3 Test Results

#### 3.3.1. Selection of Optimum Samples

The geopolymer samples in phase 2 were analyzed and after evaluating their strength, porosity, and absorption characteristics, and an optimum ratio of the blend of NaOH and KOH was selected. It was observed that 4 and 12 M combinations samples did not show comparable mechanical properties to the control samples. However, the 8 M combination samples showed comparable compressive strength, along with lesser sorptivity and absorption values than other solution samples. The two 8 M solution samples G8N_20_8K_80_ and G8N_40_8K_60_ were selected as the optimum samples and were compared to the control concrete samples.

#### 3.3.2. Slump Test

The slump values for the geopolymer concrete samples and control samples are given in Table 7. It was observed that the BM mixture had a higher slump value than the CM mixture due to the presence of sieved ground ash that caused the elimination of fibrous unburnt particles in SCBA, thus improving the workability and slump value [14]. In addition, the geopolymer samples had higher workability values than the control mixtures due to the effect of concentration and percentages of activators [46].

#### 3.3.3. Water Absorption Test

**Figure 11:**
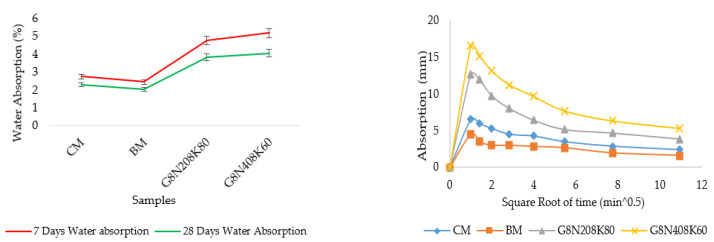

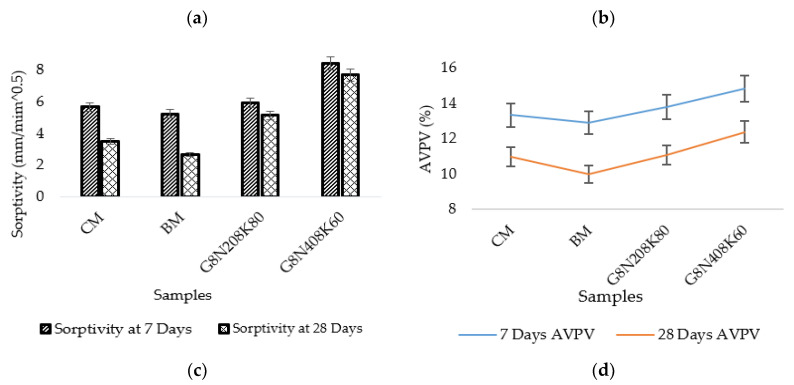
(**a**) Water absorption, (**b**) absorption rate, (**c**) sorptivity, and (**d**) AVPV of geopolymer concrete compression test.

The water absorption values of the control and optimum geopolymer samples are given in Figure 11a. It was observed that the water absorption of the control samples was less than that of geopolymer concrete samples, which was expected due to effect of ambient curing that affected the strength development and absorption rate with age. Geopolymer concrete gains strength with time when cured at ambient conditions [47]. Likewise, the water absorption of geopolymer concrete depends on the type of aluminosilicate source and particle size [48]. The effects of different dosages of SCBA aluminosilicate in geopolymer concrete still needs to be studied. Additionally, it was observed that the bagasse mixture had a lesser water absorption than the concrete mixture due to the presence of ground SCBA that filled the pore spaces of the mixture. The early age absorption of the geopolymer samples was higher due to the existence of small pores and cracks in the geopolymer structure. The percentage decrease of the geopolymer concrete samples was slightly higher than that of control samples due to the enhancement of the polymerization process along with CSH gel production.

#### 3.3.4. Sorptivity Test

The sorptivity test determined the physical features and characteristics of the concrete composite and the presence of entrained air content. The absorption rates or capillary rises of the geopolymer samples, revealing the transport mechanism of movement of water in the concrete structure, at the age of 28 days are given in Figure 11b. A comparison of the seven and 28 day sorptivity is given in Figure 11c. It can be seen that the absorption rate of CM was less than all of the samples at seven days, but at 28 days, the capillary rise of BM was less due to lesser particle size distribution of SCBA and CSH formation. However, it is evident from the following figures that the absorption rate of the control mixtures was less than that of the geopolymer concrete due to differences in their compressive strength. It can be observed that the initial absorption rate of the geopolymer concrete was higher due to the existence of dominant capillary forces. However, the secondary absorption rate was controlled by the air voids that further depended on the water to binder ratio [8]. The G8N_40_8K_60_ sample exhibited a higher absorption rate than all other mixtures due to the effect of Na^+^ and K^+^ ions in the solution samples.

#### 3.3.5. Permeable Porosity Test

The permeable porosity test results of the geopolymer concrete and control samples at seven and 28 days are given in Figure 11d. It can be observed from the figure that AVPV of the control mixture was comparatively higher than that of the geopolymer samples. Additionally, it was found that the capillary forces of the OPC concrete sample transported less water content than that of the geopolymer concrete [8]. Hence, the sustainability and durability of control mixtures in limiting water access resulted in smaller AVPV percentages. Moreover, the BM mixture at 28 days had a smaller void percentage due to the presence of ground SCBA. The high void percentage at seven days represented the incomplete strength development of samples.

**Figure 12:**
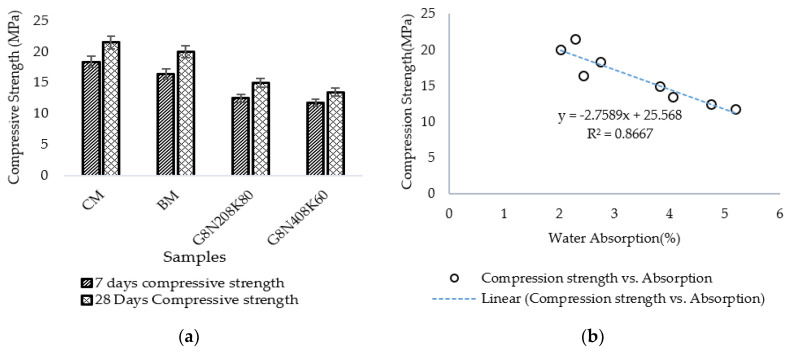
(**a**) Compressive strength of geopolymer samples at 7 and 28 days; (**b**) relationship between compression strength and water absorption.

The compression strengths of the geopolymer and control samples are given in Figure 12a. The geopolymer sample exhibited a lower strength than the control sample, which was expected due to the reaction between silicon dioxide and cement paste under the high alkaline environment in the geopolymer concrete samples. This may be attributed to the composition of SCBA composition and its particle size distribution [49]. The presence of pores and voids caused a higher absorption, resulting in a lesser compressive strength. A strong correlation existed between the water absorption and compressive strength of concrete with an R^2^ value of 0.87 (Figure 12b).

#### 3.3.6. TGA

**Figure 13:**
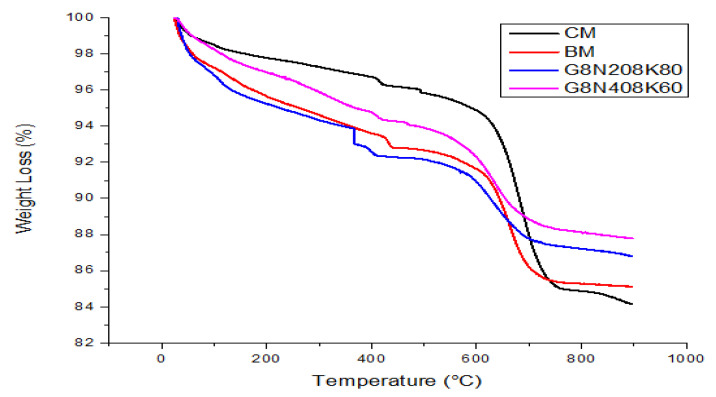
TGA of geopolymer concrete and control mixtures.

**Figure 14:**
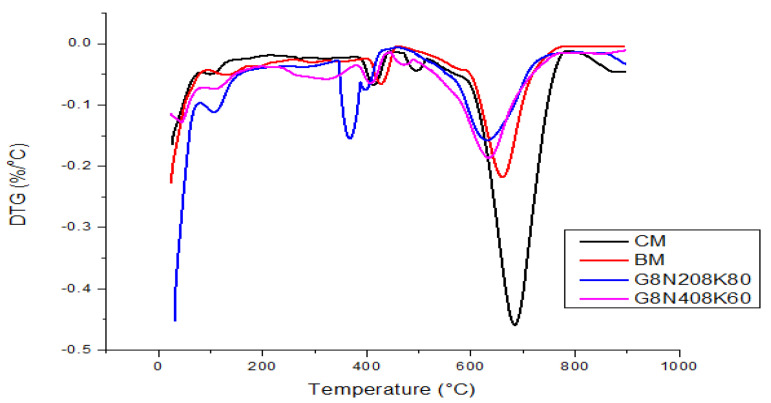
Derivative thermogravimetric (DTG) analysis of geopolymer concrete and control mixtures.

The TGA and derivative thermogravimetric (DTG) curves of two optimum geopolymer concrete (G8N_20_8K_80_ and G8N_40_8K_60_) and the two control samples (CM and BM) are presented in Figure 13 and Figure 14. Derivative curves were smoothened by the Savitzky–Golay method with a second order polynomial and 500 data points. Overall, sequence of mass loss was CM (15%) > BM (14.5%) > G8N_20_8K_80_ (12.5%) > G8N_40_8K_60_ (11.5%). The CM mixture showed a small mass loss at a lower temperature, but after 600 °C, the mass loss occurred suddenly. In the case of G8N_40_8K_60_, the mass loss at lower and higher temperatures remained low. For all four curves, before 120 °C, unbound water was evaporated along with subsequent bound water loss and the decomposition of CSH and ettringite after 120 °C. A small peak for the BM sample at nearly 190 °C was attributed to the monosulfate (AFM) phase, while a wide hump could be seen after approximately 230 °C that represented the decomposition of calcium-aluminate-hydrate (CAH) phases (C_3_AH_2_, C_4_AH_12_, and C_3_AH_6_), as shown in Figure 14 [50]. Additionally, a relatively large peak after 300 °C in the case of G8N_20_8K_80_ represented the dehydration and decomposition of hydrotalcite phases that are generally identified in alkali-activated concrete [51]. The same peak was not very pronounced in the case of G8N_40_8K_60_, which might have been due to the early dehydration of the hydrotalcite phases represented by relatively huge wide hump after 230 °C, as mentioned before. The presence of peaks between 380 and 550 °C was believed to be because of calcium hydroxide (CH) dehydration. After 600–750 °C, a huge loss of mass was due to the decarbonation of CaCO_3_.

The amount of CaCO_3_ and CH in the sample could be calculated using TG curves [52]:

(5) $$CH\;\left(\%\right)=WL\;\left(\%\right)*\;\frac{MW_{CH}}{MW_{H_{2}O}}$$

(6) $$CaCO_{3}\;\left(\%\right)=WL\;\left(\%\right)*\;\frac{MW_{CaCO_{3}}}{MW_{CO_{2}}}$$

where $MW_{CH}$ is the molecular weight of CH = 74 g/mol, $MW_{H_{2}O}$ is the molecular weight of H_2_O = 18 g/mol, $MW_{CaCO_{3}}$ is the molecular weight of CaCO_3_ = 100 g/mol, $MW_{CO_{2}}$ is the molecular weight of CO_2_ = 44 g/mol, and *WL* is the weight loss deduced from TGA curve.

The highest amount of CH was found to be 7.8%, present in the G8N_20_8K_80_ sample, which represented maximum hydration and thus depicted an earlier setting than the other samples. The CH values in the cases of CM, BM, and G8N_40_8K_60_ were 3.7%, 2.5%, and 3.3%, respectively. Surprisingly, CaCO_3_ was highest for the CM sample (21.6%), which might have been due to calcite decomposition in the raw material or by carbonation reaction, whereas it was 12.5%, 7.3%, and 9.1% for BM, G8N_20_8K_80_, and G8N_40_8K_60_, respectively.

#### 3.3.7. Acid Attack Test

**Figure 15:**
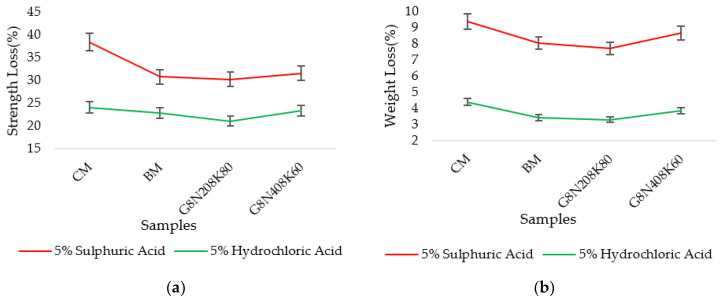
(**a**) Strength loss and (**b**) weight loss of samples.

The strength loss and weight loss in the acid attack results are tabulated in the Table 8 and Table 9, respectively, and they graphically represented in Figure 15a,b. The test results showed that geopolymer samples had less strength and weight loss than the control mixture [53]. This was due to the fact that geopolymer concrete specimens contained Na^+^ and K^+^ ions that have the propensity to produce hydrated salts when reacting with acids [54]. In addition, the deterioration of geopolymer concrete is influenced by the de-polymerization of the geopolymeric network, which in turn depends on the solubility of Si, Al, and Fe ions in strongly acidic and basic solutions [55,56]. The CM mixture had the maximum strength and weight loss. The presence of more CH in the CM mixture determined the poor performance of its acid attack [57]. However, the BM mixture had more resistance to acid attacks because the CH present in the mixture was consumed by silica, thus reducing acid attack loss [57]. Furthermore, the geopolymer concrete mixture G8N_40_8K_60_ had less resistance to acid as compared to G8N_20_8K_80_. Conversely, the strength and weight loss of the hydrochloric acid were less when compared to sulphuric acid. This was because the sulphuric acid contained a product called ettringite (calcium sulfoaluminate) that caused the disruption of concrete by its expansion [57].

#### 3.3.8. Impact of GPC on Emission Reduction and Global Warming Potential (GWP)

A life cycle assessment (LCA) involves various environmental risks related to the production and application of cement, the emission of CO_2_, the depletion of natural resources, the burning of sugarcane bagasse, the inclusion of waste ash as a cement replacement, and environmental toxicity; however, this study remained focused and limited to the emission reduction of carbon dioxide. In a typical LCA technique, four main phases are considered: (a) goal and scope phase, (b) inventory analysis phase, (c) impact assessment phase, and (d) interpretation phase, as per International Organization for Standardization ISO 14040/44 [58]. In this study, global warming potential was expressed as 100 year-embodied CO_2_-eq/m^3^. Moreover, a “cradle to mixer” approach was used to calculate the emission and comparisons with the standard control mixture. In order to simplify the analysis, the same curing conditions were considered for both the control mixture and the SCBA-based geopolymer concrete mixture. The GPC specimen showed a CO_2_ emission value of as 265 kg CO_2_ eq/m^3^, while the control mixture had CO_2_ emission value of 336 kg CO_2_ eq/m^3^. As compared to the control mixture with a 20% replacement of cement, a significant reduction in global warming potential (GWP) by around 21% was achieved. Abbas and Khereby [59] recorded a 61% reduction in GWP as compared GPC with control mixtures. However, Abbas and Khereby [59] completely replaced the cement with metakaolin.

### 3.4. Temperature Characteristics on Molar Solutions

#### 3.4.1. Sorptivity, Water Absorption, and Permeable Porosity Test Results

**Figure 16:**
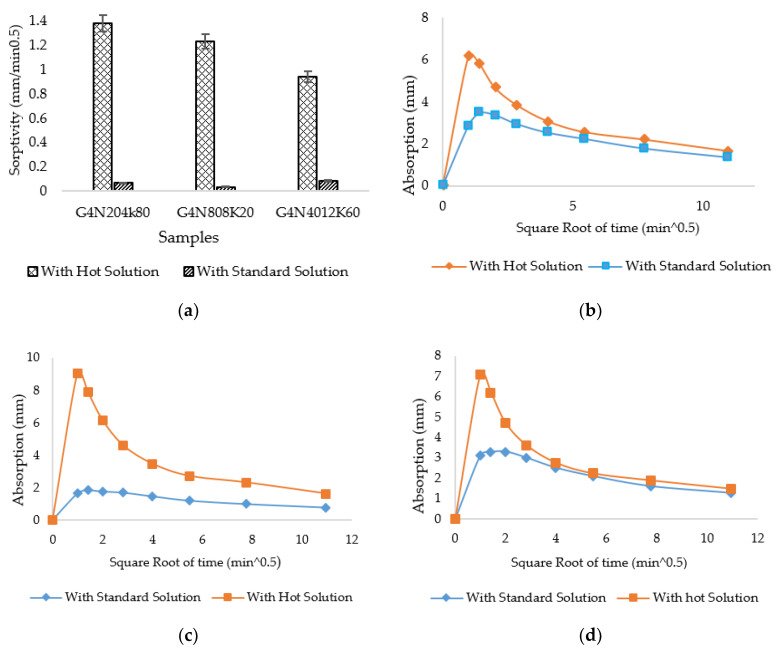
Comparison of (**a**) sorptivity values at 28 days, (**b**) absorption rate of G4N_20_4K_80_, (**c**) absorption rate of G4N_80_8K_20_, and (**d**) absorption rate of G4N_40_12K_60_.

The comparison of the sorptivity results of samples prepared with standard procedure and hot molar solution samples are presented in Figure 16a. Likewise, their absorption rates are given in Figure 16b–d. From Figure 16a, it is evident that the samples with hot solutions exhibited higher sorptivity values at both seven and 28 days. However, the percentage decrease was more significant at 28 days due to the filling of pores with age. The percentage decrease was due to the effect of the exothermic reaction of the solution when activator pellets were added in distilled water. The heat release caused small shrinkage cracks that resulted in high absorption rates.

**Figure 17:**
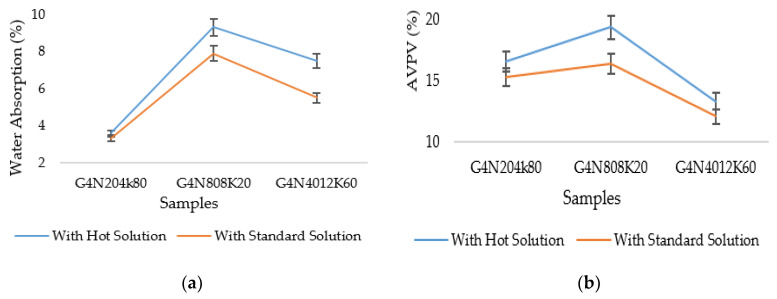
(**a**) Water absorption and (**b**) AVPV of geopolymer cement paste samples at 28 days.

In addition, the water absorption and AVPV of these geopolymer cement pastes showed the same trend as sorptivity. A comparison of water absorption values is shown in Figure 17a. It is clear that the absorption values with the standard molar solution were smaller than those of the samples prepared with the hot solutions.

On the other hand, the AVPV of the respective samples are presented in Figure 17b. The AVPV was the function of water absorption and permeable voids [8]. The percentage decrease in the AVPV of standard prepared samples represented the low percentage of pores, voids, and shrinkage cracks. The initial percentage decrease of AVPV at seven days was less than that at 28 days due to the incompletion of the CSH gel and polymerization process.

#### 3.4.2. Compressive Strength Results

**Figure 18:**
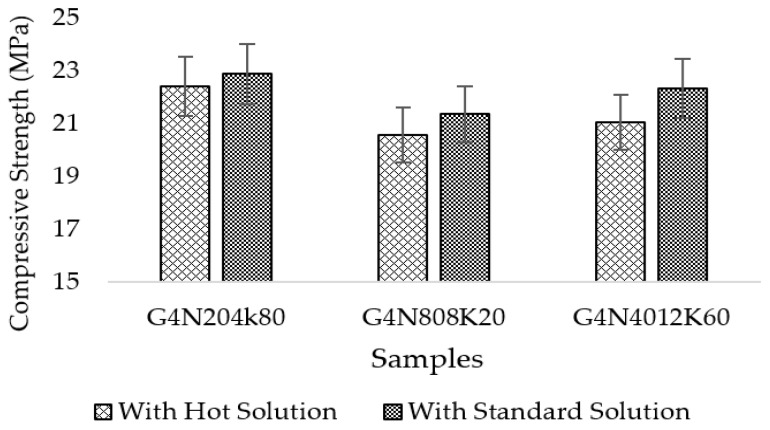
Compressive strength of geopolymer cement paste at 28 days.

The compressive strength results are given in Figure 18. The results represent the percentage increase of compressive strength of the standard prepared samples. The increase in compressive strength was due to the presence of a solid CSH phase with a lower percentage of pores. The percentage increase was obvious in the G4N_40_12K_60_ sample. Hence, it can be concluded that following standard procedure of preparation of molar solution for geopolymer concrete is compulsory. The samples prepared with the hot molar solution had poor mechanical properties.

## 4. Conclusions and Recommendation

The following conclusions can be drawn based on the above experimental investigations:

- An SCBA mixture can be used as both a pozzolan and an aluminosilicate source. From sieving and grinding, the SCBA exhibited better pozzolanic activity, as shown by its Chapelle activity. Likewise, SCBA can be used as an aluminosilicate source containing rich silica, as determined by XRD and XRF.
- The SCBA-based geopolymer cement with a higher molarity ratio had less compressive strength and higher absorption due to the presence of voids and pores caused by a vigorous exothermic reaction during the polymerization process. Furthermore, the SCBA-based geopolymer cement mixtures exhibited greater absorption and sorptivity values than the control mixtures due to the effect of temperature curing. However, the optimum geopolymer cement paste was selected from the 8 M combination solution mixture, at which better mechanical and durable properties were achieved. Thus, the two G8N_20_8K_80_ and G8N_40_8K_60_ samples were selected as the optimum samples, and a comparison between them was carried out.
- The SCBA-based geopolymer concrete had a higher workability than the control mixtures due to the effect of the concentration of NaOH and KOH activators. In addition, the water absorption and sorptivity of the SCBA-based geopolymer concrete were higher than the concrete and bagasse-based concrete mixtures due to the presence of voids and pores, as well as the alkali-aggregate effect. However, the durability performance of the geopolymer concrete was significantly better than the control cement and bagasse mixture. Furthermore, compared to the control mixture, the SCBA-based geopolymer concrete achieved a 21% reduction in global warming potential.

It is recommended to further investigate the SCBA geopolymer mixture at different percentages of OPC content, as well as to evaluate optimum temperature conditions for an SCBA-based geopolymer concrete mixture. Subsequently, a detailed investigation of life cycle assessment and costing is highly recommended. In addition, the microstructural properties of SCBA-based geopolymer concrete are still needed to be investigated.

## Figures and Tables

**Table 1:** Chemical composition of cement.

Component	Content (%)
CaO	63.34
SiO_2_	20.82
Al_2_O_3_	5.32
Fe_2_O_3_	3.27
MgO	2.56
SO_3_	2.31
K_2_O	0.98
Na_2_O	0.18
Loss of Ignition (LOI)	1.22

**Table 2:** Physical properties of aggregate.

Property	Fine Aggregate	Coarse Aggregate
Unit Weight (kg/m^3^)	1730	1610
Specific Gravity	2.62	2.65
Water Absorption (%)	0.66	0.74
Fineness Modulus	2.5	-

**Table 3:** Mix design of cement paste.

Mix	Activator to Binder Ratio (A/B)	Binder Content (g/cm^3^)	Cement (g/cm^3^)	Bagasse Ash (g/cm^3^)	Activator Content (g/m^3^)	NaOH (g/cm^3^)	KOH (g/cm^3^)
GxN_0_xk_100_	0.5	910	728	182	455	0	455
GxN_20_xk_80_	0.5	910	728	182	455	91	364
GxN_40_xk_60_	0.5	910	728	182	455	182	273
GxN_60_xk_40_	0.5	910	728	182	455	273	182
GxN_80_xk_20_	0.5	910	728	182	455	364	91
GxN_100_xk_0_	0.5	910	728	182	455	455	0

**Table 4:** Mix design of geopolymer concrete.

Mix	Activator to Binder Ratio (A/B)	Binder Content (kg/m^3^)	Cement (kg/m^3^)	Bagasse Ash (kg/m^3^)	Coarse Aggregate (kg/m^3^)	Fine Aggregate (kg/m^3^)	Activator Content (kg/m^3^)	NaOH (kg/m^3^)	KOH (kg/m^3^)
GxN_0_xk_100_	0.5	410	328	82	990	735	222	0	222
GxN_20_xk_80_	0.5	410	328	82	990	735	222	44.4	177.6
GxN_40_xk_60_	0.5	410	328	82	990	735	222	88.8	133.2
GxN_60_xk_40_	0.5	410	328	82	990	735	222	133.2	88.8
GxN_80_xk_20_	0.5	410	328	82	990	735	222	177.6	44.4
GxN_100_xk_0_	0.5	410	328	82	990	735	222	222	0

**Table 5:** Chapelle’s test results of sugar cane bagasse ash (SCBA).

Titration Volume of HCl for Control Mixture (mL) (V_1_)	Titration Volume of HCL for Ash Sample (mL) (V_2_)	Chapelle Activity = CaO (mg/gm) = [(V_1_ − V_2_)/V_1_] * 2642.86	Percent Increase w.r.t Standard (%)
9.75	4.75	1355.31	310.7

**Table 6:** XRF analysis of SCBA.

Composition	SiO_2_	Al_2_O_3_	Fe_2_O_3_	MgO	CaO	K_2_O	Na_2_O	P_2_O_5_	MnO
**Percentage (%)**	71.3	14.22	0.35	3.92	3.8	2.88	2.31	1.08	0.02

**Table 7:** Slump value of concrete samples. CM: control mixture of cement; BM: control bagasse ash mixture.

Sample Name	W/C Ratio	Slump (mm)
CM	0.5	91.44
BM	0.5	97.27
G8N_20_8K_80_	0.5	104.20
G8N_40_8K_60_	0.5	101.60

**Table 8:** Residual strength calculation after acid attack on the 28 day samples.

Mixtures	Sulphuric Acid (H_2_SO_4_)			Hydrochloric Acid (HCl)		
	Strength (P_1_) Before Test (MPa)	Strength (P_2_) After Test (MPa)	Strength Loss (%)	Strength (P_1_) Before Test (MPa)	Strength (P_2_) After Test (MPa)	Strength Loss (%)
CM	21.5	13.23	38.46	21.5	16.31	24.14
BM	20.3	13.85	30.85	20.3	15.45	22.87
G8N_20_8K_80_	15.01	10.46	30.31	15.01	11.84	21.11
G8N_40_8K_60_	13.5	9.23	31.63	13.5	10.34	23.4

**Table 9:** Residual weight loss calculation after acid attack test.

Mixtures	Sulphuric Acid(H_2_SO_4_)			Hydrochloric Acid(HCl)		
	Weight (W_1_) Before Test (gm)	Weight (W_2_) After Test (gm)	Weight Loss (%)	Weight (W_1_) Before Test (gm)	Weight (W_2_) After Test (gm)	Weight Loss (%)
CM	8340	7560	9.35	8240	7880	4.37
BM	8340	7670	8.03	8500	8210	3.41
G8N_20_8K_80_	8320	7680	7.69	8160	7893	3.27
G8N_40_8K_60_	7980	7290	8.64	8100	7790	3.82
